# Phylomitogenomics of two Neotropical species of long-legged crickets *Endecous* Saussure, 1878 (Orthoptera: Phalangopsidae)

**DOI:** 10.1590/1678-4685-GMB-2023-0144

**Published:** 2024-04-15

**Authors:** Anelise Fernandes e Silva, Henrique da Rocha Moreira Antoniolli, Edison Zefa, Vera Lúcia da Silva Valente, Maríndia Deprá

**Affiliations:** 1 Universidade Federal do Rio Grande do Sul, Departamento de Genética, Programa de Pós-Graduação em Genética e Biologia Molecular, Porto Alegre, RS, Brazil.; 2 Universidade Federal de Pelotas, Departamento de Zoologia, Ecologia e Genética, Programa de Pós-Graduação em Biodiversidade Animal, Capão do Leão, RS, Brazil.

**Keywords:** Insect, mitogenome, arrangement, Gryllidae, Phylogenetics

## Abstract

Mitochondrial genomes have provided significant insights into the evolution of several insects. A typical mitogenome contains 37 genes, and variations in gene order can indicate evolutionary relationships between species. In this study, we have assembled the first complete mitogenomes of *Endecous chape* and *E. onthophagus* and analyzed the phylogenetic implications for the Gryllidea infraorder. We performed DNA extractions and genome sequencing for both *Endecous* species. Subsequently, we searched for raw data in the Sequence Read Archive (SRA) in NCBI. Using the SRA data, we assembled the partial mitogenome of *Dianemobius nigrofasciatus* and annotated the protein-coding genes (PCGs) for nine species. Phylogenomic relationships were reconstructed using Maximum Likelihood (ML) and Bayesian Inference (BI), utilizing the PCGs from 49 Gryllidea species. The mitogenome lengths of *E. chape* and *E. onthophagus* are 16,266 bp and 16,023 bp, respectively, while *D. nigrofasciatus* has a length of 15,359 bp. Our results indicate that species within the infraorder exhibit four types of gene order arrangements that align with their phylogenetic relationships. Both phylogenomic trees displayed strong support, and the ML corroborated with the literature. Gryllidea species have significantly contributed to various fields, and studying their mitogenomes can provide valuable insights into this infraorder evolution.

## Introduction

Mitochondrial genomes can provide significant insights into organisms and genome evolution (Boore and Brown, 1998; Boore, 1999). These sequences encompass conserved genes and serve as valuable sources for genetic and molecular analysis, providing informative details on gene rearrangements, insertion, or deletion, and essential phylogenetic markers (Boore and Brown, 1998; Rokas and Holland, 2000; Fenn et al., 2008). The increased number of mitogenome sequencing has been facilitated due to their compact size, viable assembly from Next-Generation Sequencing (NGS) data, and accessible analysis in comparison to whole genomes (Fenn *et al*., 2008; Cameron, 2014; Barroso Lima and Prosdocimi, 2018).

Insects mitogenomes typically exhibit a circular structure, ranging from 14 to 19 kilobases, containing 37 genes. These genes comprise 13 protein-coding genes (PCGs), 22 transfer RNAs (tRNA), two ribosomal RNA (rRNA), and a T+A-rich region (Flook et al., 1995; Boore, 1999; Kim et al., 2005; Sheffield et al., 2010). This region is the site for replication and transcription initiation and displays high variability across insect species (Boore and Brown, 1998; Fenn et al., 2008; Gaugel et al., 2023). Mitogenomes are generally haploid with maternal inheritance and exhibit a pronounced AT-bias, characterized by a high percentage of thymine and adenine (Flook *et al*., 1995; Fenn *et al*., 2007; Erler et al., 2010; Sheffield *et al*., 2010). 

Mitochondrial phylogenomics provides a practical framework that supports evolutionary and comparative studies, including investigations into the frequency of gene rearrangements (Boore and Brown, 1998; Song et al., 2016 Cameron, 2014). In insects, diverse arrangements have been observed and that may assist in species definition. However, the full taxonomic extent of these rearrangements remains incompletely understood (Cameron, 2014).

Orthoptera has a worldwide distribution with almost 30,000 valid species, subdivided into the suborders Caelifera (grasshoppers) and Ensifera (crickets and katydids) (Song et al., 2015; Cigliano *et al*., 2023). Given the occurrence of large and complex genomes within these taxa, mitochondrial genomes have emerged as a valuable source for understanding evolution (Fenn et al., 2008). The first assembled orthopteran mitogenome was of *Locusta migratoria* (Caelifera), characterized by a 76% AT content (Flook et al., 1995). Despite limited knowledge about many groups of Orthoptera, events such as inversions, transpositions, and gene duplications and losses have been documented (Ma and Li, 2018; Yang et al., 2021; Gaugel et al., 2023).

Mitogenomes of Ensifera typically have lower AT content than those of Caelifera (Sheffield et al., 2010). Moreover, significant changes in mitochondrial gene order have been identified in Ensifera lineages, leading to the classification of distinct arrangement types (Fenn et al., 2008; Dan et al., 2022). Within the suborder Ensifera, there is the infraorder Gryllidea, encompassing the superfamilies Grylloidea (which comprises the families Gryllidae, Mogoplistidae, Trigonidiidae, Phalangopsidae, and Oechantidae) and Gryllotalpoidea (with two families, Gryllotalpidae and Myrmecophilidae) (Desutter-Grandcolas, 2003; Zhou et al., 2017; Cigliano *et al*., 2023). In phylogenetic analyses, some incongruences within the infraorder arise due to data combinations, such as the insertion of the third codon position, which can influence support values and topology (Ma and Miao, 2022).

Gryllotalpoidea mitogenomes demonstrate the preservation of ancestral gene arrangements (Yang et al., 2021). Meanwhile, within Grylloidea, two tRNA gene rearrangements are reported concerning chromosome synteny: 1) the transposition of trnV to the site between 12S and the T+A-rich region and 2) the local inversion of the ancestral trnN-trnS1-trnE to trnE-trnS1-trnN (Song et al., 2016; Yang *et al*., 2016; Zhou et al., 2017; Ma and Li, 2018). The former is described in Trigonidiidae and one Mogoplistidae species, and the second occurs in Gryllidae, Trigonidiidae, and Phalangopsidae (Ma and Li, 2018; Yang *et al*., 2021). The inversion may represent a synapomorphy of the three families, arising in a common ancestor after the divergence of Mogoplistidae (Ma and Li, 2018). 

The clade Ensifera , one of the most studied, has been employed in many studies, including cytogenetics, evolution, and embryogenesis (Donoughe and Extavour, 2016; Kataoka et al., 2022; Silva et al., 2022). Nevertheless, in Phalangopsidae, only two species possess complete mitogenomes, *Cacoplistes rogenhoferi* and *Meloimorpha japonica*, featuring the local inversion (Ma and Li, 2018).

The genus *Endecous* (Phalangopsidae) exhibits a restricted distribution, occurring only in South America (Cigliano *et al*., 2023). This clade encompasses 24 species differentiated by morphological characteristics of phallic sclerites, bioacoustics, and karyotypes, with diploid numbers ranging from 2n=14 to 21 and male sexual mechanisms of X0 or X1X20 (Zefa et al., 2014; Castro-Souza et al., 2017; Souza-Dias et al., 2017). Most *Endecous* species inhabit caves, creating gene flow between cave and surface populations, contributing significantly to cave community dynamics (Castro-Souza et al., 2017; Souza-Dias et al., 2017).

In this study, we described the first complete mitogenomes of the Phalangopsinae subfamily and compared the mitochondrial characteristics of two species of the same genus. Additionally, we analyzed gene rearrangements and inferred relationships among species of Gryllidea with available sequence data. After that, we provided the complete mitochondrial genomes for the *Endecous chape* and *Endecous onthophagus*, along with the partial mitogenome of *Dianemobius nigrofasciatus*. Obtained sequences of PCGs for nine species from raw transcriptome data available in the National Center for Biotechnology Information (NCBI). Using the 13 PCGs, we reconstructed the phylogenetic relationships of 49 species within the Gryllidea infraorder. Our results revealed that the infraorder exhibits four arrangement types, with one being unique to a single species. The analysis of mitochondrial sequences indicated that gene order rearrangements preserve phylogenetic signals, highlighting the need for deeper investigation in further studies concerning the origins and evolution of these rearrangements.

## Material and Methods

### Species samples and DNA extraction

Individuals of *E. chape* and *E. onthophagus* were provided by Laboratório de Invertebrados at Universidade Federal de Pelotas. *Endecous chape* was collected in Iguaçu National Park, Foz do Iguaçu, Paraná, Brazil (25°37’6.16”S, 54°28’56.34”W). *Endecous onthophagus* were collected in the district of Colônia Maciel, Pelotas, Rio Grande do Sul, Brazil (31º28’32”S; 52º34’09”W). The specimens were maintained in a constant temperature chamber with a 12:12h photoperiod cycle before being selected for molecular analysis. 

DNA extraction was performed using the hind femora of a female from each species with the DNeasy Blood & Tissue (Qiagen) kit following the manufacturer’s protocols. Low-coverage Next-Generation Sequencing of PE150 reads was performed by Macrogen Inc. (Seoul, South Korea) using the Illumina NovaSeq platform (Figure S1a). This generated reads with 151 bp and about 22 million and 32 million sequences (forward and reverse) for *E. chape* and *E. onthophagus*, respectively.

### Sequence analysis

*Endecous chape* and *E. onthophagus:* Raw reads underwent quality control assessment using FastQC v.0.11.9 (2019). Adapters and low-quality bases were removed using FastP tools. Mitogenome assemblies were executed using the GetOrganelle pipeline (Jin et al., 2020), employing approximately 16 million reads, resulting in a mitogenome coverage of ~1,550X for *E. chape* and 25 million reads, yielding ~1,075X of coverage for *E. onthophagus*. Then, gene annotation was conducted using Mitofinder (Allio et al., 2020) (Figure S1).

*Dianemobius nigrofasciatus:* Partial mitogenome obtained from raw data of transcriptome available in the Sequence Read Archive (SRA) of NCBI (2023) (accession number: DRR140412). Assembling was performed using the MITGARD pipeline (Nachtigall et al., 2021), with a reference mitogenome, and gene annotation was carried out with Mitofinder (Allio et al., 2020) (Figure S1b).

Nucleotide composition and the strand asymmetry (AT-Skew = (A − T)/(A + T) and GC-Skew = (G − C)/(G + C)) (Perna and Kocher, 1995) were calculated in the three mitogenomes using MEGA version 11 (Tamura et al., 2021). The asymmetry indicates the richness of the bases, then positive GC-Skew suggests an abundance of G over C, while a negative GC-Skew an excess of C over G. Similarly, a positive AT-Skew indicates an abundance of A, whereas a negative AT-Skew suggests an excess of T (Perna and Kocher, 1995).

The relative synonymous codon usage (RSCU) was determined using PhyloSuite (Zhang D et al., 2020). Synonymous codons refer to different sets of nucleotides that encode the same amino acid and may exhibit a codon usage bias, where certain codons are notably more frequent than others (Sharp et al., 1986; Subramanian et al., 2022). This is calculated by dividing the actual frequency of a codon by the frequency expected if all synonymous codons for the same amino acid were used uniformly (Sharp *et al*., 1986).

Mitochondrial DNA maps for *E. chape* and *E. onthophagus* were generated using the Circular Genome Viewer (CGView) (Stothard et al., 2019). Strand annotation was followed as described by Barroso Lima and Prosdocimi (2018), and for nucleic acid nomenclature, the IUPAC system was used.

NCBI sequences: We used 37 species of Gryllidea infraorder with complete mitochondrial genomes available in the NCBI (accessed on January 19^th^, 2023). In addition, nine species had sequences of PCGs obtained from raw data of transcriptomes available in the Sequence Read Archive (SRA) of NCBI (2023) (Table S1). The PCGs assembling and annotation were performed as it was for *D. nigrofasciatus* (described above). Except for *Marinemobius asahinai*, whose raw data came from a whole genome sequence, the assembly and annotation followed the procedures used for *Endecous* (described above) (Figure S1b).

All gene sequences were checked for the presence of nuclear copies of mitochondrial DNA (numts). They were translated and visually inspected for the presence of stop codons. Additionally, the ratio between the number of nonsynonymous substitutions per nonsynonymous sites (dN) and the number of synonymous substitutions per synonymous sites (dS) was analyzed using MEGA version 11 (Tamura et al., 2021). For dN/dS, sequences were evaluated both pairwise and overall, using the codon-based Z-test of Selection with the Nei-Gojobori method (Nei and Gojobori, 1986; Tamura *et al*., 2021). The new sequences obtained in this study have been submitted to the GenBank database, including the complete mitogenomes for *E. chape* (OQ935836) and *E. onthophagus* (OQ935837), as well as the partial mitogenome for *D. nigrofasciatus* (BK063406) (Table S1). Additionally, all annotated PCGs from the nine species used in the phylogenomic analysis are available in Table S2.

### Rearrangement analysis

The arrangements of 38 species with complete mitochondrial genomes were analyzed and classified according to the types determined by Dan et al. (2022). We used the web server Quantifying Mitochondrial Genome Rearrangement (qMGR) (Zhang J et al., 2020) to calculate the rearrangement of genes compared to a reference arrangement (benchmark). Determining the rearrangement frequency (RF) for each gene and assigning a rearrangement score (RS) to each mitogenome within a species group, evaluating them against the selected reference (Zhang J *et al*., 2020). Our reference for this analysis was the established mitochondrial gene order specific to invertebrates, available on the web server.

### Phylogenomics analysis

Inferred phylogenies were reconstructed based on the 13 PCGs from 49 species of Gryllidea and two of Tettigoniidea (*Anabrus simplex* and *Phlugiolopsis punctata*) as outgroups (Table S1). Each PCG was aligned using MAFFT v.7 (Katoh et al., 2019) and trimmed with the program TrimAl v.1.3 (Capella-Gutiérrez et al., 2009) in Phylemon2 (Sánchez et al., 2011). We conducted substitution saturation tests using DAMBE (Xia, 2017) for three codon site schemes: sites 1+2; only site 3; and all sites.

The alignments were concatenated, and the best partitioning schemes and nucleotide substitution models were estimated based on a greedy algorithm and Akaike information criteria (AIC), with PartitionFinder2 (Lanfear et al., 2017) in PhyloSuite (Zhang D et al., 2020). The gene sequences unavailable for certain species were considered missing data. Phylogenomic relationships were inferred by: (i) Maximum Likelihood (ML) analysis using IQ-Tree (Guindon et al., 2010; Nguyen et al., 2015), with an Edge-linked partition model, and 1,000 ultrafast bootstraps (UFboot) (Minh et al., 2013) replicates to estimate node supports; and (ii) Bayesian Inference (BI) analysis in MrBayes 3.2.7 (Ronquist et al., 2012) with two parallel runs of 50 million MCMC generations each, sampling every 5,000 and discarding the first 25% samples as burn-in.

## Results

### Mitogenome and sequence characterizations

**Figure 1 -:**
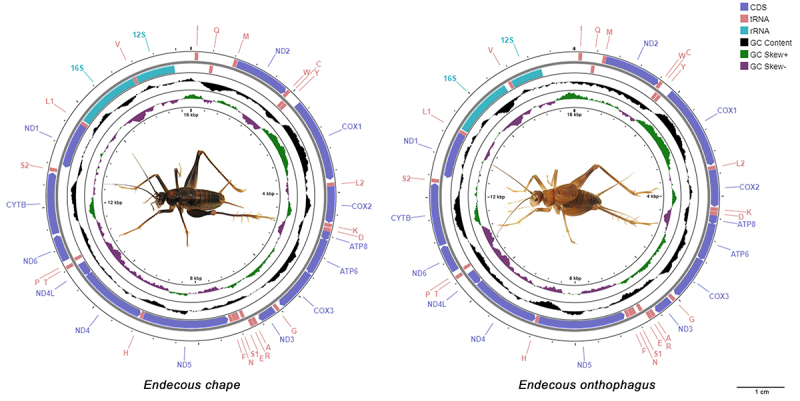
Complete mitochondrial genome maps of *Endecous chape* and *E. onthophagus*. Representative picture of specimens. Legend indicates CDs (Protein-coding genes), RNAs (transfer RNA), and rRNAs (ribosomal RNA) using the IUPAC system of nomenclature.

The mitogenome lengths for *E. chape* and *E. onthophagus* were 16,266 bp and 16,023 bp, respectively. They have the usual gene content, with 13 PCGs, two rRNAs, and 22 tRNAs genes (Figure 1). The 13 PCGs are compounds by two ATPase subunits (Atp6 and Atp8), the cytochrome C oxidase subunits COI, COII, and COIII, one cytochrome b gene (CYTB), and seven NADH dehydrogenase subunits (ND1-6 and ND4l). The ribosomal RNAs 12S and 16S, and the 22 tRNAs, the trnA, trnR, trnN, trnD, trnC, trnQ, trnE, trnG, trnH, trnI, trnL1, trnL2, trnK, trnM, trnF, trnP, trnS1, trnS2, trnT, trnW, trnY, and trnV (Figure 1 and Table S3). The partial genome of *D. nigrofasciatus*, assembled using raw data from NCBI, comprises 15,359 bp, including all PCGs and all rRNAs, but lacks tRNA-Glutamic acid (trnE) as indicated in Table S3.

The PCGs displayed substantial size diversity, from 162 - 171 bp in ATP8 to 1,725 - 1,734 bp in ND5. Regarding rRNAs, sizes varied between 627 - 785 bp in 12S and 1,193 - 1,375 bp in 16S. Similarly, the 22 tRNAs exhibited a consistent length range of 48 - 71 bp (Table S3).

*Endecous chape*, *E. onthophagus*, and *D. nigrofasciatus* mitogenomes showed a GC content ranging from 27.3% to 29.3% (Table 1). When analyzing each mitochondrial gene, the GC content was consistently lower than the AT content (Table 1). According to the nucleotide composition, most genes displayed a higher percentage of thymine than adenine (Table S3).

**Table 1 -:** Percentage of AT and GC, and the AT-Skew and GC-Skew of the mitochondrial genome of *Dianemobius nigrofasciatus*, *Endecous chape*, and *E. onthophagus* and each gene.

Mitogenome	AT%	GC%	AT-Skew	GC-Skew
^a^ *D. nigrofasciatus*	71.5	27.3	-0.011	-0.331
*E. chape*	72.4	27.6	0.044	-0.332
*E. onthophagus*	70.7	29.3	0.063	-0.345
^b^Genes				
ATP6	71.6/69.5/68.4	28.3/30.4/31.6	-0.131/-0.113/-0.073	-0.392/-0.356/-0.398
ATP8	79.0/76.0/74.6	21.0/24.0/25.4	0.062/0.000/-0.041	-0.412/-0.707/-0.714
COI	65.7/65.4/64.8	34.3/34.6/35.3	-0.146/-0.080/-0.093	-0.131/-0.126/-0.137
COII	70.1/67.6/68.9	29.9/32.5/31.2	-0.012/-0.026/-0.054	-0.269/-0.300/-0.289
COIII	68.9/66.8/64.3	31.1/33.2/35.8	-0.147/-0.131/-0.057	-0.208/-0.221/-0.241
CytB	68.2/68.0/65.3	31.9/31.9/34.8	-0.135/-0.129/-0.078	-0.260/-0.286/-0.288
NAD1	71.8/72.6/68.7	28.2/27.5/31.3	-0.235/-0.298/-0.348	0.402/0.347/0.399
NAD2	74.5/75.8/75.3	25.4/24.2/24.7	-0.213/-0.066/-0.082	-0.392/-0.410/-0.377
NAD3	75.1/70.4/71.9	24.8/29.7/28.1	-0.203/-0.148/-0.123	-0.409/-0.340/-0.283
NAD4	73.8/72.9/72.2	26.3/27.1/27.8	-0.147/-0.254/-0.258	0.455/0.455/0.432
NAD4L	79.0/73.6/72.1	21.0/26.4/27.9	-0.174/-0.291/-0.337	0.770/0.644/0.688
NAD5	74.2/74.1/71.4	25.8/25.9/28.6	-0.134/-0.206/-0.289	0.434/0.367/0.431
NAD6	75.2/77.7/73.4	24.7/22.4/26.6	-0.098/-0.105/-0.032	-0.554/-0.418/-0.597
12S	71.0/69.9/68.4	29.0/30.1/31.6	-0.006/-0.056/-0.072	0.385/0.314/0.384
16S	74.4/67.9/72.7	25.7/32.2/27.3	-0.013/-0.049/-0.119	0.457/0.292/0.405
tRNAs(L)	75.3/75.5/75.6	24.7/24.5/24.4	0.041/0.065/0.077	0.051/0.056/0.022
tRNAs(H)	72.4/73.2/72.5	27.6/26.8/27.4	-0.018/-0.073/-0.028	0.422/0.374/0.363

^a^ The mitochondrial genome is incomplete. ^b^Data of genes are given as *D. nigrofasciatus/ E. chape*/ *E. onthophagus*. tRNAs: concatenated tRNA genes. (L) light strand. (H) heavy strand.

Concerning strand asymmetry, the AT-skew values for *E. chape* and *E. onthophagus* (0.044 and 0.063) were positive and negative for *D. nigrofasciatus* (-0.011), and all showed negative values of GC-skew (-0.345 to -0.332) (Table 1). Most of the PCGs showed negative AT-skew, except ATP8 in *D. nigrofasciatus* (0.062) and *E. chape* (0.000), and GC-skew also has negative values, except for the genes ND1, ND4, ND4l, and ND5. The two rRNAs showed negative AT-skew and positive GC-skew values. For tRNAs, the light strand has AT-skew and GC-skew positive, and the heavy AT-skew negative and GC-skew positive (Table 1).

In all three species, most PCGs start with ATN codons, except for COI, which starts with CAA in *D. nigrofasciatus* and ACG *in E. onthophagus*. Additionally, ND1 consistently starts with TTG across all three mitogenomes (Table S3). Concerning stop codons, the predominant one is TAA, yet variations exist, such as TAG found in CytB and ND3 of *D. nigrofasciatus*, as well as in ND4l of *E. onthophagus* (Table S3).

We recovered and annotated the PCGs for *Acheta domesticus*, *Gryllus assimilis*, *G. firmus*, *G. pennsylvanicus*, *G. rubens*, *G. texensis*, *M. asahinai*, *Phaeophilacris bredoides*, and *Teleogryllus commodus* (Table S2). Mitogenome length ranged from 15,791 bp in *M. asahinai* to 16,066 bp in *G. pennsylvanicus* (Table S1).

The saturation tests were applied in each aligned gene with all species to analyze codon site 1+2, only site 3, and with all the sites, considering significant values of p>0.05. In the test on-site 3, most sequences showed high saturation and were classified as useless. In the test on sites 1+2, the genes ATP6, COII, COIII, ND1, ND3, and ND6 showed p<0.05 and little saturation. Then, for the other genes, the best results were observed in the test involving all codon positions (Table S4).

**Figure 2 -:**
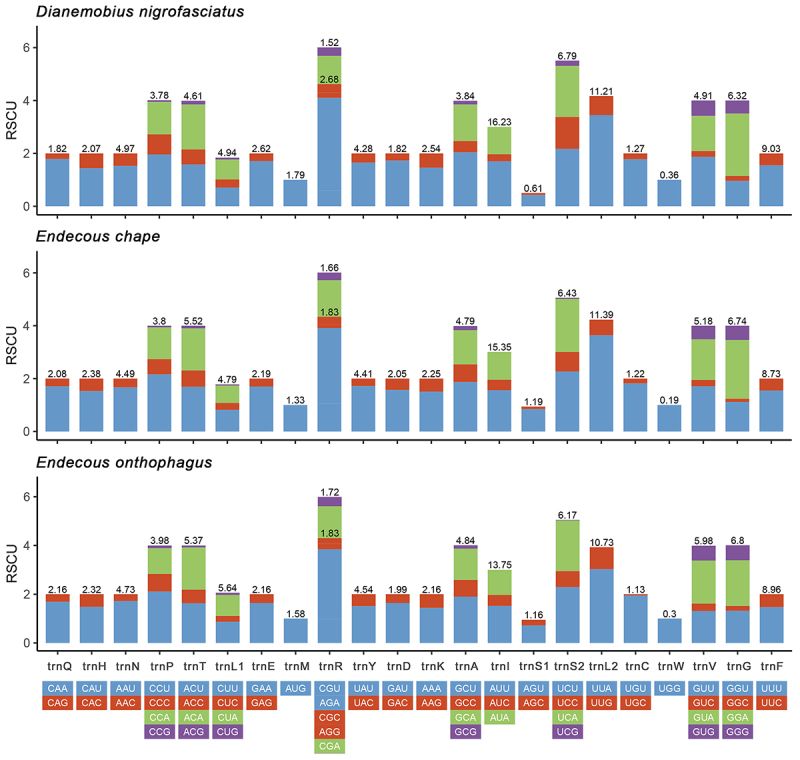
Relative synonymous codon usage **(**RSCU) of *Dianemobius nigrofasciatus*, *Endecous chape*, and *E. onthophagus* mitogenomes. The numbers above the colored columns indicate the frequencies of amino acids in the mitochondrial genomes.

The relative synonymous codon usage (RSCU) of both *Endecous* and *D. nigrofasciatus* showed similar values (Figure 2). The codons most frequently used for de amino acids are trnL2 (UUA), trnS2 (UCU), trnR (AGA), and trnG (GGA), and the trnP (CCU) and trnS2 (UCA) only in *E. chape* and *E. onthophagus*. These codons showed RSCU values higher than 2.00 (Figure 2 and Table S5).

### Rearrangements

**Figure 3 -:**
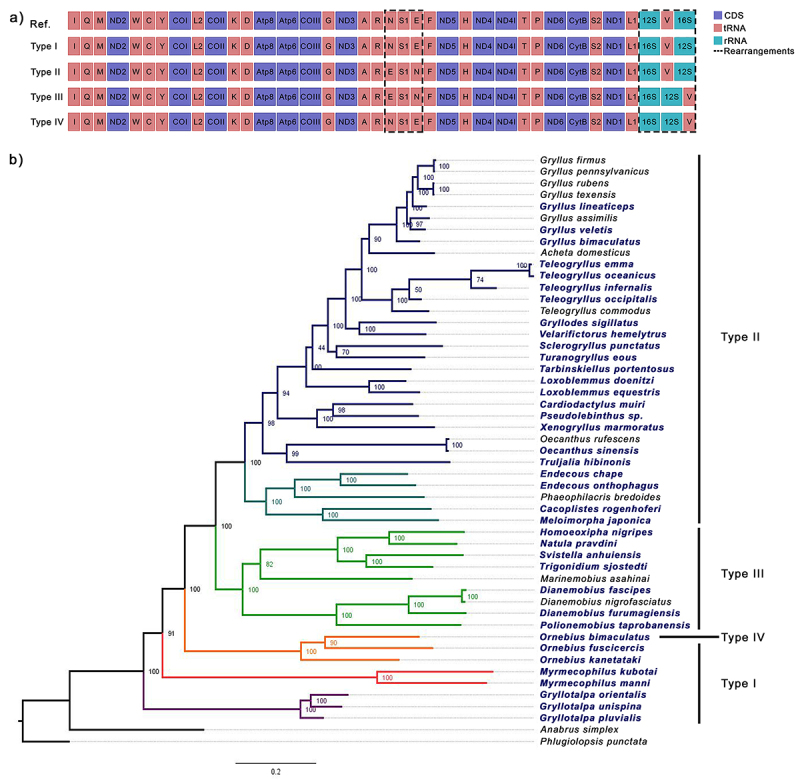
Representation of mitochondrial gene rearrangements in Gryllidea species, distribution, and relationship in the Maximum Likelihood (ML). a) Gene order of reference (Ref.) mitogenome and arrangement types. Legend indicates the CDs (Protein-coding genes), RNAs (transfer RNA), rRNAs (ribosomal RNA), and dot line rearrangement’s location. b) Phylogenomic tree inferred with the 13 PCGs, values next to the nodes indicate the bootstrap support. Each branch color indicates the species family: purple Gryllotalpidae, orange Mogoplistidae, red Myrmecophilidae, green Trigonidiidae, dark green Phalangopsidae, and dark blue Gryllidae. Species names in bold/blue have their mitogenome complete and were used to analyze the rearrangements.

We identified four arrangements from 38 complete mitogenomes analyzed and compared these gene orders with the mitochondrial gene order established for invertebrates available on the web server qMGR (Zhang J et al., 2020) (Figure 3). Type I occurs in seven species within the subfamilies Gryllotalpidae, Myrmecophilidae, and Mogoplistidae. We observed modification in the typical gene order of 12S-trnV-16S to 16S-trnV-12S. The group Type II consists of 23 species that belong to Gryllidae and Phalangopsidae. Compared with the reference mitogenome, it shows an inversion of the gene order trnN-trnS1-trnE to trnE-trnS1-trnN. Seven species of Trigonidiidae were classified in Type III and showed the inversion trnE-trnS1-trnN and the modification of 12S-V-16S to 16S-12S-trnV. Finally, the Type IVG (Gryllidea infraorder) occurred only in the scaly cricket *Ornebius bimaculatus* and consisted of the 16S-12S-trnV (Figure 3a ). 

The total rearrangement frequency (RF) and rearrangement score (RS) were calculated for each gene and the groups of arrangements, respectively (Figure S2). We found the highest RF value for 16S (100), followed by 12S and trnV (89.47), then the trnN, trnS1, and trnE with 78.94. All the PCGs showed RF equal to zero, so they have the same position as the typical invertebrate mitogenome (Figure S2a). The RS test indicated that Type II is the most active rearrangement with a score of 16, followed by Type III with 14, Type I with eight, and Type IVG with six (Figure S2b).

### Phylogenomic reconstructions

The best nucleotide substitution model was GTR+I+G for all genes, and the partitioning scheme clustered them in eleven partitions (ND1, ND2, ND3, ND5+ND4, ATP6, ATP8+ND6, COI, COII, COIII, CYTB, ND4l). According to the saturation test, we obtained good results for codon positions 1+2 and all sites. Then, for the genes Atp6, COII, COIII, ND1, ND3, and ND6, we selected only the codon sites 1+2 (Table S4).

The ML and BI consensus trees exhibited variations in species relationships and displayed high node support (Figure 3b  and Figure S3, respectively). Species of the same genus were consistently clustered in both analyses, indicating their close relationships. The first discrepancy between the results is the position of the clade containing *Myrmecophilus* and *Ornebius*; however, the BI had stronger support (PP = 1.00 and PP = 0.9). Subsequently, in the BI tree, *Tarbinskiellus portentosus* showed a close relationship with *Loxoblemmus* (PP = 1.00). Following, while the ML tree showed a close relationship between *Sclerogryllus punctatus* and *Turanogryllus eous* (UFboot = 70), this association was not corroborated by the BI analysis (PP = 1.00 and PP = 0.92, respectively). Finally, the different placements of *Teleogryllus occipitalis* and *T. commodus*, with better support in the BI tree (Figure 3b  and Figure S3).

## Discussion

### Mitogenomes and characterizations

We sequenced and characterized the complete mitogenomes of *E. chape* and *E. onthophagus*, with lengths of 16,266 bp and 16,023 bp, respectively. They contain 37 genes (13 PCGs, 22 tRNAs, and two rRNAs) and represent the first fully assembled mitogenomes for the Phalangopsinae subfamily. Additionally, we obtained the partial mitogenome of *D. nigrofasciatus*, with 15,359 bp and 36 genes, lacking the trnE. This is the third characterized assembly for the *Dianemobius* genus (Ma et al., 2019).

The mitogenomes assembled and inferred in this study showed sizes similar to those typically observed in insects (Flook et al., 1995; Boore, 1999; Kim et al., 2005; Sheffield et al., 2010). The length of complete mitogenomes among Phalangopsidae species varies from 15,880 bp in *M. japonica* to 16,266 bp in *E. chape* (Ma and Li, 2018).

The PCGs consistently feature the standard ATN start codons, except for COI in *D. nigrofasciatus* and *E. onthophagus*, as well as ND1 in *E. chape*, *E. onthophagus,* and *D. nigrofasciatus*. Similar variations in COI start codons have been observed in several species such as *C. rogenhoferi*, *M. japonica*, *Ornebius fuscicercis*, and others (Ma and Li, 2018). Likewise, ND1 variations have been noted in *C. rogenhoferi*, *M. japonica*, and *Polionemobius taprobanensis* (Ma and Li, 2018; Ma *et al*., 2019).

The use of synonymous codons to encode most amino acids often displays a bias towards specific codons over others. This selective preference can be influenced by different factors, including nucleotide composition (Subramanian et al., 2022). Species with genomes rich in AT content typically favor codons ending in A/T, such as *Gryllotalpa orientalis*, where 79.2% of the third codon position ends in A/T (Kim et al., 2005; Dan et al., 2022; Subramanian *et al*., 2022). Furthermore, a higher frequency of A/T-ending genes positively impacts the AT bias of mitogenomes (Ma and Miao, 2022).

The amino acids most frequently used in *Endecous* and *D. nigrofasciatus* are Leucine (UUA) and Serine (UCU), which are common among other orthopterans. In *G. orientalis*, these amino acid frequencies were 15.79% and 9.58%, respectively (Kim et al., 2005). However, these findings slightly differ from the commonly considered most frequent amino acids in other Ensifera mitogenomes, including Isoleucine (AUU), Phenylalanine (UUU), and Leucine (UUA) (Ma and Miao, 2022; Dan et al., 2022). Regarding the methionine tRNA, we did not observe either duplications or deletions. 

All three cricket mitogenomes exhibit strong GC-skew values ranging from -0.345 to -0.332, a characteristic shared with species of Eneopterinae and Gryllinae (Ensifera) (Dong et al., 2017). These negative GC skew values indicate an excess of C relative to G in these mitogenomes (Dong *et al*., 2017; Ma et al., 2019). The AT-skew values ranged between *Endecous* (0.044 and 0.063) and *D. nigrofasciatus* (-0.011). Despite these differences, the values found for *Endecous* align with those observed in Phalangopsidae, while those for *D. nigrofasciatus* are according to Nemobiinae (Ma *et al*., 2019). So, it is possible to infer the presence of an A excess in Phalangopsidae and of T in Nemobiinae (Ma *et al*., 2019).

### Rearrangements

Type I through Type IVG arrangements have previously been documented in Ensifera species (Kim et al., 2005; Ma and Li, 2018; Dan et al., 2022). Type I, Type II, and Type III were previously classified by Dan *et al*. (2022). However, the Type IVG rearrangement, exclusively found in *O. bimaculatu*s had not been categorized before (Ma and Li, 2018; Dan et al., 2022) therefore, we categorize it for the first time in this study.

The Type I classification encompasses *G. orientalis*, *G. pluvialis* of Gryllotalpinae, and *Myrmecophilus manni* of Myrmecophilinae (Dan et al., 2022). Expanding this group are *G. unispina*, *M. kubotai*, *O. fuscicercis,* and *O. kanetataki* of the Mogoplistinae subfamily, which was not included previously (Ma and Li, 2018; Dan *et al*., 2022; Ma and Miao, 2022). Regarding the standard gene order in invertebrates, both Caelifera and Ensifera exhibit different rearrangements. In Ensifera, Type I shows the rearrangement 16S-trnV-12S, whereas species of Caelifera, such as *L. migratoria*, exhibit a translocation of tRNAs K and D (Flook et al., 1995; Kim et al., 2005; Dan *et al*., 2022). Consequently, it can be inferred that the observed rearrangements in Caelifera and Ensifera evolved independently after the split of the Orthoptera stem lineage (Kim *et al*., 2005; Cameron et al., 2006).

Type II shows the inversion trnN-trnS1-trnE to trnE-trnS1-trnN (Figure 3a ), and it is most prevalent among the described complete mitogenomes (Ma and Li, 2018; Dan et al., 2022). The inversion observed in *C. rogenhoferi* and *M. japonica* was also found in both *Endecous* species, confirming the position of the Phalangopsidae as a Type II (Ma and Li, 2018). Additionally, we added *S. punctatus* of the Sclerogryllinae subfamily to this group (Yu et al., 2022). Type II has been proposed to occur in the Gryllinae species, although it might be a feature of the Gryllidae family (Yang et al., 2016). It is necessary to examine a larger number of samples to correctly characterize the inversion and determine its prevalence among species.

Type III comprises the species described by Dan et al. (2022) from the Nemobiinae and Trigonidiinae subfamilies (Ma et al., 2019). Lastly, Type IVG found exclusively in *O. bimaculatus*, stands as a distinct category within the Gryllidea infraorder (Ma and Li, 2018). This type is characterized by featuring the typical trnN-trnS1-trnE gene order and the transposition of trnV to the site between 12S and the T+A-rich region (Ma and Li, 2018). The trnV relocation is also observed in Trigonidiidae, implying that it occurred independently in these two branches (Gaugel et al., 2023). Moreover, Type IVG differs from Type IV described by Dan *et al*. 2022, which occurs only in *Comicus campestris* (Tettigoniidea) and involves the deletion of trnI.

Concerning the total rearrangement score (RS), our analysis revealed a decrease of Type II > Type III > Type I > Type IVG, which could reflect the number of described mitogenomes. Type II includes seven subfamilies covering 23 species, Type III includes seven species across two subfamilies, Type I encompasses seven species from three subfamilies, and Type IVG contains a single species. The RS of each mitogenome within a species group can be applied as rearrangement features for comparative studies and may provide a reference for comprehending rearrangement evolution. Furthermore, each rearrangement frequency (RF) is crucial for understanding evolutionary dynamics within a group (Zhang J et al., 2020; Zhang *et al*., 2021). Our findings emphasize the importance of additional research on Gryllidea including species of different clades and from the same genus, which may reveal new rearrangements, as demonstrated in Ma and Li’s (2018) study on *Ornebius*.

### Phylogenomics

The phylogenomic analysis recovered *E. chape* and *E. onthophagus* as sister species, closely related to *P. bredoides*. This clade clustered with *C. rogenhoferi* and *M. japonica*, positioning them all within Phalangopsidae. This relationship was expected since the first three belong to Phalangopsinae, and both *C. rogenhoferi* and *M. japonica* to Cachoplistinae (Cigliano *et al*., 2023).

Within Gryllidae, certain correlations were confirmed, including the close relationship between the genus *Oecanthus* and *Truljalia hibinonis*, and *Gryllodes sigillatus* and *V. hemelytrus* (Ma et al., 2019; Yang et al., 2021; Dan et al., 2022; Ma and Miao, 2022). The close relationship between *A. domesticus* and *Gryllus* was also supported, consistent with previous studies involving *A. domesticus* and *G. bimaculatus* (Ma and Miao, 2022).

The species *Gryllus rubens* and *G. texensis* are closely related, as are *G. firmus* and *G. pennsylvanicus*, and they still interact creating hybrid zones within the USA. The first two species diverged around 500,000 years ago, and COI and transcriptome-based SNP data analysis revealed that a bidirectional gene flow persisted until approximately 18,000 years ago (Blankers et al., 2018; Kataoka et al., 2022). In the case of *G. firmus* and *G. pennsylvanicus*, their divergence occurred about 200,000 years ago, and gene flow continues at the present (Maroja et al., 2009; Larson et al., 2013; Kataoka *et al*., 2022).

The cluster containing *Ornebius* has shown variations in previous phylogenetic inferences according to the method applied (Ma et al., 2019; Yang et al., 2021; Yu et al., 2022). However, our analysis strongly supported the clustering of *O. fuscicercis* and *O. bimaculatus*, with *O. kanetataki* as their sister group. 

In our study, we observed discrepancies between the ML and BI analyses, and as previously observed, the BI trees exhibited higher support (Ma and Miao, 2022). However, one discrepancy involves the positioning of the *Myrmecophilus* and *Ornebius* genera, where the ML tree was consistent with findings from previous studies (Yang et al., 2021; Dan et al., 2022; Ma and Miao, 2022; Gaugel et al., 2023). Incorporating the third codon position might lead to more robust tree topologies (Ma and Miao, 2022). Therefore, its selective inclusion across certain genes could have influenced the phylogenomic relationships.

## Conclusion

Phylogenetic investigations have confirmed that the distribution of gene rearrangements aligns with phylogenetic relationships. Our findings indicate that the four observed rearrangements may be a distinctive feature related to phylogenomic relationships. It is crucial to continue examining mitogenomes to verify the consistent occurrence of these rearrangements and to uncover potential new variations, as exemplified by the unique case of *O. bimaculatus*. Additionally, species within Gryllidea hold significant ecological and social importance, making them ideal model organisms for diverse fields of study.

## Supplementary material

The following online material is available for this article:

Table S1 - Information on the investigated species.

Table S2 - Protein-coding genes recovered and annotated for phylogenetic analysis.

Table S3 - Length and nucleotide composition of the mitogenome genes.

Table S4 - Saturation test.

Table S5 - Codon usage analysis of mitogenomes.

Figure S1 **-**
Scheme of new data origin and methodology to obtain mitochondrial sequences.

Figure S2 **-**
Rearrangements analysis with the web server qMGR.

Figure S3 - Bayesian Inference (BI) tree of infraorder Gryllidea using the 13 PCGs.
