# Supplementary material for: Phylomitogenomics of two Neotropical species of long-legged crickets Endecous Saussure, 1878 (Orthoptera: Phalangopsidae)
Source: Genet Mol Biol. 2024 Apr 15;46(3 Suppl 1):e20230144. doi: 10.1590/1678-4685-GMB-2023-0144 (PMC11034622; doi:10.1590/1678-4685-GMB-2023-0144)
Supplement: Table S1 - [file 1415-4757-GMB-46-03-s1-e20230144-s1.pdf]

Genetics and Molecular Biology

https://doi.org/10.1590/1678-4685-GMB-2023-0144

Supplementary Material to "Phylomitogenomics of two Neotropical species of long-legged crickets *Endecous* Saussure, 1878 (Orthoptera: Phalangopsidae)"

Table S1 - Information on the investigated species, including mitochondrial genome sizes and NCBI access numbers.

Taxa	Species	Size (bp)	Accession number	Reference
**Superfamily Grylloidea**				
**Gryllidae**				
Eneopterinae	*Cardiodactylus muiri* Otte, 2007	16,328	NC037914	Dong et al., 2017
	Pseudolebinthus sp. Robillard, 2006	16,075	MN414243	Salazar et al., 2020
	Xenogryllus marmoratus (Haan, 1844)	15,762	NC041236	Ma et al., 2019b
Gryllinae	*Acheta domesticus* (Linnaeus, 1758)	16,003	SRR2230498	Zhou et al., 2018
	Gryllodes sigillatus (Walker, 1869)	16,369	NC057195	Yang et al., 2021
	Gryllus assimilis (Fabricius, 1775)	16,050	SRR10619397	Submitted by Harvard University (2019)
	Gryllus bimaculatus De Geer, 1773	16,075	MT993975	Park et al., 2021
	Gryllus firmus Scudder, 1902	16,029	SRR835515	Nanoth Vellichirammal et al., 2014
	Gryllus lineaticeps Stål, 1861	15,607	NC057052	Torson et al., 2022
	Gryllus pennsylvanicus Burmeister, 1838	16,066	SRR3987008	Submitted by University of Western Ontario (2016)
	Gryllus rubens Scudder, 1902	16,010	SRR3182700	Submitted by Museum fuer Naturkunde (2016)
	Gryllus texensis Cade & Otte, 2000	16,017	SRR6761203	Submitted by Museum fuer Naturkunde (2018)
	Gryllus veletis (Alexander & Bigelow, 1960)	15,686	MW322713	Torson et al., 2022
	Loxoblemmus doenitzi Stein, 1881	15,396	NC033985	Zhou, 2016
	Loxoblemmus equestris Saussure, 1877	16,314	KU562919	Yang et al., 2016
	Tarbinskiellus portentosus (Lichtenstein, 1796)	15,710	BK059220	Homchan and Gupta, 2022

Taxa	Species	Size (bp)	Accession number	Reference
	Teleogryllus commodus (Walker, 1869)	15,598	SRR1424310	Submitted by University of New South Wales (2014)
	Teleogryllus emma (Ohmachi & Matsuura, 1951)	15,660	NC011823	Ye et al., 2008
	Teleogryllus infernalis (Saussure, 1877)	15,512	MK903574	Chang et al., 2020
	Teleogryllus occipitalis (Serville, 1838)	15,501	LC521855	Kataoka et al., 2020
	Teleogryllus oceanicus (Le Guillou, 1841)	15,660	NC028619	Zhou et al., 2017a
	Turanogryllus eous Bey-Bienko, 1956	16,045	NC060317	Ma et al., 2019c
	Velarifictorus hemelytrus (Saussure, 1877)	16,123	NC030762	Yang et al., 2016
Oecanthinae	*Oecanthus rufescens* Serville, 1838	15,617	KX057720	Zhou et al., 2017b
	Oecanthus sinensis Walker, 1869	16,142	NC034799	Li et al., 2019
Podoscirtinae	*Truljalia hibinonis* (Matsumura, 1917)	15,120	NC034797	Li et al., 2019
Sclerogryllinae	*Sclerogryllus punctatus* (Brunner von Wattenwyl, 1893)	15,438	OL875084	He, 2021
**Mogoplistidae**				
Mogoplistinae	*Ornebius bimaculatus* (Shiraki, 1930)	16,136	NC039666	Ma and Li, 2018
	Ornebius fuscicercis (Shiraki, 1930)	16,368	NC039739	Ma and Li, 2018
	Ornebius kanetataki (Matsumura, 1904)	16,589	NC039667	Ma and Li, 2018
**Phalangopsidae**				
Cachoplistinae	*Cacoplistes rogenhoferi* Saussure, 1877	16,018	NC039664	Ma and Li, 2018
	Meloimorpha japonica (Haan, 1844)	15,880	NC039665	Ma and Li, 2018
Phalangopsinae	*Endecous chape* Souza-Dias & de Mello, 2017	16,266	OQ935836	This study
	Endecous onthophagus (Berg, 1891)	16,023	OQ935837	This study
	Phaeophilacris bredoides Kaltenbach, 1986	16,085	SRR1811982	Wipfler et al., 2019
**Trigonidiidae**				
Nemobiinae	*Dianemobius fascipes* (Walker, 1869)	15,363	NC045846	Ma et al., 2019a
	Dianemobius furumagiensis (Ohmachi & Furukawa, 1929)	15,350	NC045847	Ma et al., 2019a
	[a]*Dianemobius nigrofasciatus* (Matsumura, 1904)	15,359	BK063406	This study
	Marinemobius asahinai (Yamasaki, 1979)	15,791	DRR300664	Submitted by Department of Evolutionary Studies of Biosystems,

Taxa	Species	Size (bp)	Accession number	Reference
	Polionemobius taprobanensis (Walker, 1869)	16,641	NC045848	SOKENDAI (The Graduate University for Advanced Studies) (2021) Ma et al., 2019a
Trigonidiinae	*Homoeoxipha nigripes* Xia & Liu, 1993	15,679	NC045841	Ma et al., 2019a
	Natula pravdini (Gorochov, 1985)	15,817	NC050742	Ma and Li, 2017
	Svistella anhuiensis He, Li & Liu, 2009	16,494	NC053543	Ma and Li, 2017
	Trigonidium sjostedti (Chopard, 1925)	15,763	NC032077	Song et al., 2016
**Superfamily Gryllotalpoidea**				
**Gryllotalpidae**				
Gryllotalpinae	*Gryllotalpa orientalis* Burmeister, 1838	15,521	AY660929	Kim et al., 2005
	Gryllotalpa pluvialis (Mjöberg, 1913)	15,525	EU938371	Fenn et al., 2008
	Gryllotalpa unispina Saussure, 1874	15,513	NC029148	Wang et al., 2013
**Myrmecophilidae**				
Myrmecophilinae	*Myrmecophilus kubotai* Maruyama, 2004	15,345	MZ440658	Sanno et al., 2021
	Myrmecophilus manni Schimmer, 1911	15,323	NC011301	Fenn et al., 2008
**Superfamily Tettigonioidea**				
**Tettigoniidae**				
Tettigoniinae	*Anabrus simplex* Haldeman, 1852	15,766	EF373911	Fenn et al., 2007
Meconematinae	*Phlugiolopsis punctata* Wang, Li & Liu, 2012	17,461	NC068775	Liang and Bian, 2022

[a]Partial mitochondrial genome obtained from raw data with access number DRR140412 (Submitted by Animal Physiology, Graduate School of Science, Osaka City University (2020)).

References

Chang H, Qiu Z, Yuan H, Wang X, Li X, Sun H, ... and Huang, Y (2020) Evolutionary rates of and selective constraints on the mitochondrial genomes of Orthoptera insects with different wing types. Molecular phylogenetics and evolution 145:106734.

Dong J, Vicente N, Chintauan-Marquier I, Ramadi C, Dettai A and Robillard T. (2017) Complete mitochondrial genome and taxonomic revision of *Cardiodactylus muiri* Otte, 2007 (Gryllidae: Eneopterinae: Lebinthini). Zootaxa 4268:101-116.

Fenn JD, Cameron SL and Whiting MF (2007) The complete mitochondrial genome sequence of the Mormon cricket (*Anabrus simplex*: Tettigoniidae: Orthoptera) and an analysis of control region variability. Insect molecular biology 16:239-252.

Fenn JD, Song H, Cameron SL and Whiting MF (2008) A preliminary mitochondrial genome phylogeny of Orthoptera (Insecta) and approaches to maximizing phylogenetic signal found within mitochondrial genome data. Molecular Phylogenetics and Evolution 49:59-68.

He ZQ (2021) Submitted (15-DEC-2021) School of Life Sciences, East China Normal University, 500 Dongchuan Road, Shanghai, Shanghai 200241, China.

Homchan S and Gupta YM (2022) The complete mitochondrial genome of giant cricket, *Tarbinskiellus portentosus* (Orthoptera: Gryllidae) and its curation. Mitochondrial DNA Part B 7:1427-1431.

Kataoka K, Minei R, Ide K, Ogura A, Takeyama H, Takeda M, ... and Asahi T (2020) The draft genome dataset of the Asian cricket *Teleogryllus occipitalis* for molecular research toward entomophagy. Frontiers in Genetics 11:470.

Kim I, Cha SY, Yoon MH, Hwang JS, Lee SM, Sohn HD, and Jin BR (2005) The complete nucleotide sequence and gene organization of the mitochondrial genome of the oriental mole cricket, *Gryllotalpa orientalis* (Orthoptera: Gryllotalpidae). Gene 353:155-168.

Li J, Chen Q, Wen M, Wang J, Wang Y and Ren B (2019) Phylogeny and acoustic signal evolution of a pure tone song katydid *Pseudophyllus titan* (Orthoptera: Tettigoniidae) based on the complete mitogenome. Mitochondrial DNA Part A 30:385-396.

Liang L and Bian X (2022) Submitted (01-MAR-2022) Guangxi Normal University, Key Laboratory of Ecology of Rare and Endangered Species and Environmental Protection (Guangxi Normal University), Yanzhong Road, Guilin, GuangXi 541000, China.

Ma C and Li J (2017) Mitochondrial genome sequence and gene rearrangement provide insights into the taxonomic affiliation of sword-tailed crickets. Submitted (17-DEC-2017) Institute of Apicultural Research, Chinese Academy of Agricultural Sciences, Beijing, China

Ma C and Li J (2018) Comparative analysis of mitochondrial genomes of the superfamily Grylloidea (Insecta, Orthoptera) reveals phylogenetic distribution of gene rearrangements. International journal of biological macromolecules 120:1048-1054.

Ma C, Wang Y, Zhang L and Li J (2019) Mitochondrial genome characterization of the family Trigonidiidae (Orthoptera) reveals novel structural features and nad1 transcript ends. Scientific Reports 9:19092.

Ma C, Zhang L and Li J (2019) Characterization of the complete mitochondrial genome of a bush cricket *Xenogryllus marmoratus* (Insecta: Orthoptera). Mitochondrial DNA Part B 4:172-173.

Ma C, Zhang L and Li J (2019) The complete mitochondrial genome of a field cricket *Turanogryllus eous* (Insecta: Orthoptera). Mitochondrial DNA Part B 4:3852-3853.

Nanoth Vellichirammal N, Zera AJ, Schilder RJ, Wehrkamp C, Riethoven JJM and Brisson JA (2014) De novo transcriptome assembly from fat body and flight muscles transcripts to identify morph-specific gene expression profiles in *Gryllus firmus*. PloS one 9:e82129.

Park B, Choi EH, Kim G, Shin CR, Hwang J, Baek SY and Hwang UW (2021) The complete mitochondrial genome of the two-spotted cricket *Gryllus bimaculatus* (Orthoptera: Gryllidae) from South Korea. Mitochondrial DNA Part B 6:1144-1146.

Salazar K, Murphy RJ, Guillaume M, Nattier R and Robillard T (2020) *Pseudolebinthus lunipterus* sp. nov.: a striking deaf and mute new cricket from Malawi (Orthoptera, Gryllidae, Eneopterinae). PeerJ 8:8204.

Sanno R, Kataoka K, Hayakawa S, Ide K, Nguyen CN, Nguyen TP, Le BTN, Kim OTP, Mineta K, Takeyama H, Takeda M, Sato T, Suzuki T, Yura K and Asahi T (2021) Submitted (24-JUN-2021) Advanced Science and Engineering, WASEDA University, Wakamatsu 2-2, Shinjuku, Tokyo 185-8480, Japan.

Song N, Li H, Song F and Cai W (2016) Molecular phylogeny of Polyneoptera (Insecta) inferred from expanded mitogenomic data. Scientific Reports 6:36175.

Torson AS, Hicks AM, Baragar CE, Smith DR and Sinclair BJ (2022) The mitochondrial genomes of two *Gryllus* crickets (Grylloidea: Gryllidae) via RNA-seq. Mitochondrial DNA Part B 7:106-107.

Wang P, Zhi Y and Zhang D (2013) Sequencing and Analysis of *Gryllotalpa unispina* Mitochondrial Genome. Submitted (11-APR-2013) College of Life Sciences, Hebei University, 180 Wusidong Road, Baoding, Hebei 071002, China.

Wipfler B, Letsch H, Frandsen PB, Kapli P, Mayer C, Bartel D, ... and Simon S (2019) Evolutionary history of Polyneoptera and its implications for our understanding of early winged insects. Proceedings of the National Academy of Sciences 116:3024-3029.

Yang J, Dong H, He M and Gao J (2021) Mitochondrial genome characterization of *Gryllodes sigillatus* (Orthoptera: Gryllidae) and its phylogenetic implications. Mitochondrial DNA Part B 6:1056-1058.

Yang J, Ren Q and Huang Y (2016) Complete mitochondrial genomes of three crickets (Orthoptera: Gryllidae) and comparative analyses within Ensifera mitogenomes. Zootaxa 4092:529-547.

Ye W, Dang JP, Xie LD and Huang Y (2008) Complete mitochondrial genome of *Teleogryllus emma* (Orthoptera: Gryllidae) with a new gene order in Orthoptera.

Zhou ZJ (2016) Towards a higher-level phylogeny of ensiferan insects inferred from mitochondrial genome sequences. Submitted (06-APR-2016) College of Life Sciences, Hebei University,No. 180 Wusidong Road, Baoding, Hebei 071002, China

Zhou C, Liu S, Song W, Luo S, Meng G, Yang C, ... and Zhou X (2018) Characterization of viral RNA splicing using whole-transcriptome datasets from host species. Scientific reports 8:3273.

Zhou JX, Jia YC, Yang XC and Li Q (2017) The complete mitochondrial genome of the black field cricket, *Teleogryllus oceanicus*. Mitochondrial DNA Part A 28:229-230.

Zhou Z, Zhao L, Liu N, Guo H, Guan B, Di J and Shi F (2017) Towards a higher-level Ensifera phylogeny inferred from mitogenome sequences. Molecular Phylogenetics and Evolution 108:22-33.
