# Supplementary material for: Phylomitogenomics of two Neotropical species of long-legged crickets Endecous Saussure, 1878 (Orthoptera: Phalangopsidae)
Source: Genet Mol Biol. 2024 Apr 15;46(3 Suppl 1):e20230144. doi: 10.1590/1678-4685-GMB-2023-0144 (PMC11034622; doi:10.1590/1678-4685-GMB-2023-0144)
Supplement: Table S2 - [file 1415-4757-GMB-46-03-s1-e20230144-s2.pdf]

Genetics and Molecular Biology

https://doi.org/10.1590/1678-4685-GMB-2023-0144

Supplementary Material to "Phylomitogenomics of two Neotropical species of long-legged crickets *Endecous* Saussure, 1878 (Orthoptera: Phalangopsidae)"

Table S2 – Protein-coding genes that have been recovered and annotated for phylogenetic analysis, along with their respective NCBI accession numbers.

Species	Atp6	Atp8	COI	COII	COIII	CytB	ND1	ND2	ND3	ND4	ND4l	ND5	ND6
Acheta domesticus	-	-	-	-	BK063533	-	BK063534	-	-	-	-	-	-
Gryllus assimilis	BK063535	BK063536	BK063537	BK063538	BK063539	BK063540	-	BK063541	BK063542	BK063543	BK063544	BK063545	-
Gryllus firmus	BK063546	BK063547	BK063548	BK063549	-	-	BK063550	-	BK063551	BK063552	BK063553	BK063554	-
Gryllus pennsylvanicus	-	-	-	-	-	-	-	-	-	BK063555	-	-	-
Gryllus rubens	BK063556	BK063557	BK063558	BK063559	BK063560	BK063561	BK063562	-	BK063563	BK063564	BK063565	BK063566	-
Gryllus texensis	BK063567	BK063568	BK063569	BK063570	BK063571	BK063572	BK063573	-	BK063574	BK063575	BK063576	BK063577	-
Phaeophilacris bredoides	-	-	BK063578	-	-	-	-	-	-	-	BK063579	-	-
Teleogryllus commodus	BK063580	-	BK063581	BK063582	BK063583	BK063584	-	-	-	-	BK063585	-	-

Species	Atp6	Atp8	COI	COII	COIII	CytB	ND1	ND2	ND3	ND4	ND4l	ND5	ND6
Marinemobius asahinai	BK063586	-	BK063587	BK063588	BK063589	BK063590	BK063591	BK063592	BK063593	BK063594	BK063595	BK063596	BK063597

(-) Missing data.
