# Supplementary material for: Phylomitogenomics of two Neotropical species of long-legged crickets Endecous Saussure, 1878 (Orthoptera: Phalangopsidae)
Source: Genet Mol Biol. 2024 Apr 15;46(3 Suppl 1):e20230144. doi: 10.1590/1678-4685-GMB-2023-0144 (PMC11034622; doi:10.1590/1678-4685-GMB-2023-0144)
Supplement: Table S3 - [file 1415-4757-GMB-46-03-s1-e20230144-s3.pdf]

Genetics and Molecular Biology

https://doi.org/10.1590/1678-4685-GMB-2023-0144

Supplementary Material to "Phylomitogenomics of two Neotropical species of long-legged crickets *Endecous* Saussure, 1878 (Orthoptera: Phalangopsidae)"

Table S3 – Length and nucleotide composition of the mitogenome genes of *Dianemobius nigrofasciatus, Endecous chape*, and *E. onthophagus*.

Mitogenomes	Size (bp)	T%	C%	A%	G%		
[a]*D. nigrofasciatus*	15,359	36.6	18.4	35.8	9.2		
E. chape	16,266	34.6	18.4	37.8	9.2		
E. onthophagus	16,023	33.1	19.7	37.6	9.6		
[b]**Genes**						**Start codon**	**Stop codon**
Atp 6	684/684/684	40.5/38.7/36.7	19.7/20.6/22.1	31.1/30.8/31.7	8.6/9.8/9.5	ATG/ATG/ATG	TAA/TAA/TAA
Atp 8	162/171/165	37.0/38.0/38.8	14.8/20.5/21.8	42.0/38.0/35.8	6.2/3.5/3,6	ATT/ATA/ATA	TAA/TAA/TAA
COI	1,536/1,542/1,536	37.6/35.3/35.4	19.4/19.5/20.1	28.1/30.1/29.4	14.9/15.1/15.2	CAA/ATA/ACG	TAA/TAA/TAA
COII	696/678/678	35.5/34.7/36.3	19.0/21.1/20.1	34.6/32.9/32.6	10.9/11.4/11.1	ATG/ATG/ATG	TAA/---/---
COIII	789/789/789	39.5/37.8/34.0	18.8/20.3/22.2	29.4/29.0/30.3	12.3/12.9/13.6	ATG/ATG/ATG	TAA/TAA/TAA

Mitogenomes	Size (bp)	T%	C%	A%	G%		
CYTB	1,137/1,140/1,140	38.7/38.4/35.2	20.1/20.5/22.4	29.5/29.6/30.1	11.8/11.4/12.4	ATG/ATG/ATG	TAG/TAA/TAA
ND1	936/915/915	44.3/47.1/46.3	8.4/9.0/9.4	27.5/25.5/22.4	19.8/18.5/21.9	TTG/TTG/TTG	---/---/---
ND2	1,020/1,035/1,041	45.2/40.4/40.7	17.7/17.1/17.0	29.3/35.4/34.6	7.7/7.1/7.7	ATT/ATT/ATT	TAA/TAA/TAA
ND3	354/327/234	45.2/40.4/40.6	17.5/19.9/17.9	29.9/30.0/32.1	7.3/9.8/9.4	ATT/ATT/ATT	TAG/TAA/TAA
ND4	1,341/1,341/1,341	42.3/45.7/45.4	7.2/7.4/7.9	31.5/27.2/26.8	19.1/19.7/19.9	ATG/ATG/ATG	---/---/---
ND4L	291/276/276	46.447.5/48.2	2.4/4.7/4.3	32.6/26.1/23.9	18.6/21.7/23.6	ATG/ATA/ATG	TAA/TAA/TAG
ND5	1,725/1,731/1,734	42.1/44.7/46.0	7.3/8.2/8.1	32.1/29.4/25.4	18.5/17.7/20.5	ATT/ATA/ATA	---/---/---
ND6	489/492/504	41.3/42.9/37.9	19.2/15.9/21.2	33.9/34.8/35.5	5.5/6.5/5.4	ATT/ATT/ATA	TAA/TAA/TAA
12S	762/785/627	35.7/38.7/36.7	8.9/8.5/9.7	35.3/33.8/31.7	20.1/19.0/21.9		
16S	1,306/1,375/1,193	37.7/38.3/40.7	7.0/7.4/8.1	36.7/35.0/32.0	18.7/19.3/19.2		
Ala	65/69/65	40.0/39.1/40.0	9.2/10.1/12.3	36.9/39.1/36.9	13.8/11.6/10.8		
Arg	61/66/66	34.4/28.8/33.3	13.1/18.2/13.6	39.3/39.4/40.9	13.1/13.6/12.1		
Asn	58/64/64	34.5/35.9/32.8	15.5/9.4/10.9	29.3/42.2/42.2	20.7/12.5/14.1		
Asp	66/67/71	40.9/43.3/42.3	6.1/4.5/5.6	48.5/46.3/47.9	4.5/6.0/4.2		
Cys	62/64/62	35.5/39.1/38.7	11.3/7.8/8.1	38.7/35.9/35.5	14.5/17.2/17.7		
Gln	69/69/69	44.9/44.9/44.9	5.8/10.1/8.7	30.4/29.0/29.0	18.8/15.9/17.4		
Glu	-/67/69	-/46.3/42.0	-/4.5/5.8	-/41.8/40.6	-/7.5/11.6		

Mitogenomes	Size (bp)	T%	C%	A%	G%
Gly	63/63/67	36.5/34.9/34.3	12.7/9.5/10.4	41.3/39.7/44.8	9.5/15.9/10.4
His	64/65/67	39.1/43.1/37.3	4.7/6.2/4.5	35.9/30.8/34.3	20.3/20.0/23.9
Ile	66/66/67	37.9/31.8/35.8	9.1/9.1/10.4	39.4/43.9/38.8	13.6/15.2/14.9
Leu1	66/67/69	33.3/34.3/31.9	6.1/7.5/7.2	34.8/38.8/39.1	25.8/19.4/21.7
Leu2	64/67/65	34.4/38.8/35.4	9.4/9.0/9.2	34.4/37.3/41.5	21.9/14.9/13.8
Lys	70/70/70	34.3/32.9/28.6	17.1/14.3/18.6	35.7/38.6/37.1	12.9/14.3/15.7
Met	68/69/69	33.8/31.9/31.9	17.6/18.8/18.8	35.3/33.3/33.3	13.2/15.9/15.9
Phe	64/66/68	35.9/31.8/32.4	4.7/7.6/7.4	40.6/36.4/39.7	18.8/24.2/20.6
Pro	64/64/63	37.5/40.6/38.1	6.3/7.8/7.9	35.9/28.1/31.7	20.3/23.4/22.2
Ser1	65/68/66	30.8/39.7/39.4	12.3/13.2/15.2	41.5/27.9/25.8	15.4/19.1/19.7
Ser2	48/64/66	29.2/35.9/34.8	8.3/7.8/9.1	43.8/42.2/43.9	18.8/14.1/12.1
Thr	62/66/65	38.7/36.4/35.4	9.7/12.1/9.2	38.7/39.4/41.5	12.9/12.1/13.8
Trp	65/67/66	35.4/34.3/31.8	12.3/13.4/13.6	40.0/43.3/40.9	12.3/9.0/13.6
Tyr	65/66/67	35.4/39.4/34.3	10.8/6.1/9.0	32.3/34.8/37.3	21.5/19.7/19.4
Val	67/68/68	40.3/27.9/38.2	7.5/23.5/11.8	35.8/36.8/32.4	16.4/11.8/17.6

[a]The mitochondrial genome is incomplete. [b]Data of genes are given as *D. nigrofasciatus/ E. chape/ E. onthophagus*. (-) Missing data.
