# Supplementary material for: Phylomitogenomics of two Neotropical species of long-legged crickets Endecous Saussure, 1878 (Orthoptera: Phalangopsidae)
Source: Genet Mol Biol. 2024 Apr 15;46(3 Suppl 1):e20230144. doi: 10.1590/1678-4685-GMB-2023-0144 (PMC11034622; doi:10.1590/1678-4685-GMB-2023-0144)
Supplement: Table S4 - [file 1415-4757-GMB-46-03-s1-e20230144-s4.pdf]

Genetics and Molecular Biology

https://doi.org/10.1590/1678-4685-GMB-2023-0144

Supplementary Material to "Phylomitogenomics of two Neotropical species of long-legged crickets *Endecous* Saussure, 1878 (Orthoptera: Phalangopsidae)"

Table S4 – Saturation test. a) Shows the number of operational units (NumOTU); index of substitutional saturation (Iss); critical index of substitutional saturation assuming a symmetrical topology (Iss.cSym); p-value; critical index of substitutional saturation assuming an asymmetrical topology (Iss.cAsym).

Codon position	NumOTU	Iss	Iss.cSym	T	DF	P	Iss.cAsym	T	DF	P
Atp6 1+2	4	0.286	0.792	19.656	381	0.0000	0.759	18.363	381	0.0000
	8	0.284	0.747	16.607	381	0.0000	0.635	12.599	381	0.0000
	16	0.289	0.712	14.502	381	0.0000	0.503	7.333	381	0.0000
	32	0.292	0.697	13.676	381	0.0000	0.370	2.619	381	0.0092
Atp6 3	4	0.696	0.777	4.550	224	0.0220	0.762	1.888	224	0.0603
	8	0.734	0.732	3.558	224	0.9572	0.631	3.084	224	0.0023
	16	0.739	0.653	2.756	224	0.0063	0.458	9.052	224	0.0000
	32	0.745	0.685	2.034	224	0.0431	0.364	12.914	224	0.0000
Atp6	4	0.423	0.806	17.165	603	0.0000	0.775	15.774	603	0.0000
	8	0.421	0.767	14.626	603	0.0000	0.658	9.999	603	0.0000
	16	0.421	0.747	13.519	603	0.0000	0.537	4.838	603	0.0000
	32	0.427	0.72	12.064	603	0.0000	0.395	1.319	603	0.1876
Atp8 1+2	4	0.509	0.816	5.105	85	0.0000	0.871	6.01	85	0.0000
	8	0.520	0.817	4.690	85	0.0000	0.777	4.044	85	0.0001
	16	0.522	0.581	0.916	85	0.3625	0.500	0.349	85	0.7280
	32	0.534	0.840	4.802	85	0.0000	0.648	1.795	85	0.0762
Atp8 3	4	0.834	0.962	1.610	44	0.1145	1.127	3.682	44	0.0006
	8	0.812	1.071	3.300	44	0.0019	1.147	4.270	44	0.0001
	16	0.811	0.560	3.354	44	0.0016	0.722	1.190	44	0.2403
	32	0.820	1.266	6.220	44	0.0000	1.381	7.838	44	0.0000
Atp8	4	0.581	0.785	4.162	130	0.0001	0.801	4.486	130	0.0000
	8	0.602	0.757	3.043	130	0.0028	0.681	1.535	130	0.1272
	16	0.612	0.610	0.046	130	0.9634	0.459	3.032	130	0.0029
	32	0.613	0.735	2.459	130	0.0152	0.460	3.075	130	0.0026
COI 1+2	4	0.149	0.821	48.708	753	0.0000	0.789	46.389	753	0.0000
	8	0.146	0.789	43.679	753	0.0000	0.684	36.519	753	0.0000
	16	0.161	0.772	39.633	753	0.0000	0.575	26.846	753	0.0000
	32	0.160	0.749	38.216	753	0.0000	0.445	18.467	753	0.0000
COI 3	4	0.662	0.796	5.736	505	0.0000	0.762	4.281	505	0.0000
	8	0.679	0.752	3.263	505	0.0012	0.641	1.691	505	0.0915
	16	0.688	0.722	1.618	505	0.1063	0.513	8.333	505	0.0000
	32	0.694	0.703	0.445	505	0.6563	0.378	15.791	505	0.0000
COI	4	0.311	0.834	36.965	1274	0.0000	0.804	34.845	1274	0.0000
	8	0.314	0.810	32.538	1274	0.0000	0.710	26.004	1274	0.0000
	16	0.307	0.793	31.382	1274	0.0000	0.611	19.594	1274	0.0000
	32	0.320	0.776	29.018	1274	0.0000	0.494	11.043	1274	0.0000
COII 1+2	4	0.284	0.792	21.855	387	0.0000	0.759	20.427	387	0.0000
	8	0.274	0.747	19.776	387	0.0000	0.635	15.101	387	0.0000
	16	0.275	0.711	18.209	387	0.0000	0.502	9.468	387	0.0000
	32	0.291	0.696	17.362	387	0.0000	0.369	3.349	387	0.0009
COII 3	4	0.703	0.777	2.069	221	0.0397	0.762	1.668	221	0.0968
	8	0.745	0.733	0.361	221	0.7185	0.631	3.278	221	0.0012
	16	0.766	0.652	3.478	221	0.0006	0.458	9.447	221	0.0000

	32	0.784	0.686	3.183	221	0.0017	0.365	13.553	221	0.0000
COII	4	0.417	0.806	18.307	612	0.0000	0.775	16.844	612	0.0000
	8	0.413	0.767	15.799	612	0.0000	0.657	10.912	612	0.0000
	16	0.409	0.746	15.144	612	0.0000	0.536	5.736	612	0.0000
	32	0.430	0.720	13.130	612	0.0000	0.394	1.645	612	0.1005
COIII 1+2	4	0.244	0.797	26.191	453	0.0000	0.764	24.608	453	0.0000
	8	0.255	0.754	21.913	453	0.0000	0.642	17.025	453	0.0000
	16	0.262	0.725	20.584	453	0.0000	0.515	11.257	453	0.0000
	32	0.286	0.705	18.787	453	0.0000	0.378	4.136	453	0.0000
COIII 3	4	0.728	0.778	1.593	255	0.1124	0.758	0.955	255	0.3406
	8	0.741	0.732	0.297	255	0.7666	0.627	3.813	255	0.0002
	16	0.763	0.666	3.596	255	0.0004	0.464	11.005	255	0.0000
	32	0.788	0.682	4.177	255	0.0000	0.356	17.075	255	0.0000
COIII	4	0.399	0.811	20.837	693	0.0000	0.780	19.258	693	0.0000
	8	0.413	0.775	17.715	693	0.0000	0.667	12.421	693	0.0000
	16	0.422	0.757	16.373	693	0.0000	0.551	6.304	693	0.0000
	32	0.431	0.730	14.61	693	0.0000	0.411	0.960	693	0.3373
CytB 1+2	4	0.304	0.810	25.433	623	0.0000	0.779	23.866	623	0.0000
	8	0.318	0.773	20.840	623	0.0000	0.664	15.868	623	0.0000
	16	0.331	0.754	18.582	623	0.0000	0.547	9.491	623	0.0000
	32	0.357	0.728	15.805	623	0.0000	0.406	2.093	623	0.0367
CytB 3	4	0.764	0.786	0.833	370	0.4053	0.756	0.283	370	0.7777
	8	0.767	0.740	1.029	370	0.3041	0.629	5.276	370	0.0000
	16	0.797	0.697	4.116	370	0.0000	0.488	12.740	370	0.0000
	32	0.822	0.689	5.559	370	0.0000	0.359	19.315	370	0.0000
CytB	4	0.430	0.825	22.443	964	0.0000	0.793	20.626	964	0.0000
	8	0.474	0.795	17.066	964	0.0000	0.690	11.513	964	0.0000
	16	0.482	0.778	15.464	964	0.0000	0.584	5.345	964	0.0000
	32	0.508	0.756	12.855	964	0.0000	0.458	2.625	964	0.0088
ND1 1+2	4	0.269	0.800	25.384	493	0.0000	0.769	23.861	493	0.0000
	8	0.295	0.759	20.976	493	0.0000	0.648	15.977	493	0.0000
	16	0.306	0.734	19.389	493	0.0000	0.523	9.852	493	0.0000
	32	0.314	0.710	18.238	493	0.0000	0.382	3.141	493	0.0018
ND1 3	4	0.701	0.780	2.543	284	0.0115	0.756	1.782	284	0.0757
	8	0.721	0.733	0.423	284	0.6728	0.626	3.180	284	0.0016
	16	0.730	0.675	1.954	284	0.0516	0.470	9.213	284	0.0000
	32	0.752	0.682	2.638	284	0.0088	0.354	14.999	284	0.0000
ND1	4	0.407	0.815	21.794	777	0.0000	0.784	20.108	777	0.0000
	8	0.422	0.780	18.384	777	0.0000	0.673	12.883	777	0.0000
	16	0.428	0.763	17.159	777	0.0000	0.560	6.774	777	0.0000
	32	0.437	0.738	15.61	777	0.0000	0.424	0.673	777	0.5011
ND2 1+2	4	0.516	0.803	12.349	589	0.0000	0.771	10.999	589	0.0000
	8	0.511	0.762	10.361	589	0.0000	0.652	5.811	589	0.0000
	16	0.526	0.739	8.943	589	0.0000	0.528	0.082	589	0.9349
	32	0.537	0.714	7.582	589	0.0000	0.386	6.484	589	0.0000

ND2 3	4	0.855	0.781	2.527	306	0.0120	0.756	3.403	306	0.0008
	8	0.876	0.734	5.393	306	0.0000	0.626	9.530	306	0.0000
	16	0.874	0.680	8.279	306	0.0000	0.474	17.059	306	0.0000
	32	0.886	0.683	9.497	306	0.0000	0.354	24.904	306	0.0000
ND2	4	0.618	0.817	10.606	893	0.0000	0.786	8.912	893	0.0000
	8	0.622	0.784	8.394	893	0.0000	0.677	2.860	893	0.0043
	16	0.630	0.767	7.273	893	0.0000	0.566	3.408	893	0.0007
	32	0.641	0.742	5.598	893	0.0000	0.432	11.559	893	0.0000
ND3 1+2	4	0.439	0.777	9.437	224	0.0000	0.761	8.988	224	0.0000
	8	0.490	0.732	5.940	224	0.0000	0.630	3.423	224	0.0007
	16	0.519	0.656	3.356	224	0.0009	0.460	1.450	224	0.1484
	32	0.520	0.684	3.994	224	0.0001	0.362	3.834	224	0.0002
ND3 3	4	0.827	0.793	0.709	116	0.4800	0.821	0.134	116	0.8933
	8	0.874	0.773	2.205	116	0.0294	0.707	3.657	116	0.0004
	16	0.911	0.599	7.137	116	0.0000	0.469	10.113	116	0.0000
	32	0.928	0.763	3.820	116	0.0002	0.512	9.639	116	0.0000
ND3	4	0.583	0.784	6.386	338	0.0000	0.756	5.472	338	0.0000
	8	0.589	0.738	4.601	338	0.0000	0.628	1.191	338	0.2344
	16	0.621	0.691	2.077	338	0.0385	0.483	4.134	338	0.0000
	32	0.647	0.686	1.159	338	0.2473	0.356	8.723	338	0.0000
ND4 1+2	4	0.537	0.816	14.209	701	0.0000	0.784	12.596	701	0.0000
	8	0.577	0.782	10.236	701	0.0000	0.674	4.890	701	0.0000
	16	0.619	0.764	7.158	701	0.0000	0.562	2.828	701	0.0048
	32	0.652	0.739	4.134	701	0.0000	0.426	10.802	701	0.0000
ND4 3	4	0.822	0.791	1.197	441	0.2319	0.758	2.504	441	0.0127
	8	0.858	0.746	4.431	441	0.0000	0.634	8.866	441	0.0000
	16	0.919	0.710	8.828	441	0.0000	0.501	17.688	441	0.0000
	32	0.938	0.696	10.729	441	0.0000	0.368	25.245	441	0.0000
ND4	4	0.650	0.830	11.329	1206	0.0000	0.798	9.352	1206	0.0000
	8	0.666	0.803	8.366	1206	0.0000	0.701	2.157	1206	0.0312
	16	0.672	0.786	6.895	1206	0.0000	0.599	4.427	1206	0.0000
	32	0.705	0.767	3.758	1206	0.0002	0.477	13.835	1206	0.0000
ND4l 1+2	4	0.369	0.777	11.129	179	0.0000	0.774	11.04	179	0.0000
	8	0.380	0.738	9.149	179	0.0000	0.645	6.778	179	0.0000
	16	0.383	0.634	6.496	179	0.0000	0.453	1.820	179	0.0705
	32	0.393	0.698	8.040	179	0.0000	0.392	0.028	179	0.9775
ND4l 3	4	0.743	0.817	1.338	88	0.1842	0.873	2.345	88	0.0213
	8	0.765	0.82	1.024	88	0.3085	0.780	0.283	88	0.7780
	16	0.787	0.58	4.035	88	0.0001	0.502	5.556	88	0.0000
	32	0.798	0.844	0.949	88	0.3453	0.655	2.943	88	0.0042
ND4l	4	0.481	0.779	9.121	268	0.0000	0.757	8.466	268	0.0000
	8	0.499	0.732	6.797	268	0.0000	0.626	3.712	268	0.0002
	16	0.499	0.668	4.998	268	0.0000	0.466	0.972	268	0.3319
	32	0.511	0.680	5.124	268	0.0000	0.355	4.661	268	0.0000
ND5 1+2	4	0.459	0.824	20.811	982	0.0000	0.792	18.990	982	0.0000

	8	0.457	0.794	18.506	982	0.0000	0.690	12.783	982	0.0000
	16	0.472	0.778	16.70	982	0.0000	0.584	6.126	982	0.0000
	32	0.479	0.756	15.221	982	0.0000	0.457	1.184	982	0.2365
ND5 3	4	0.787	0.799	0.545	556	0.5860	0.767	0.878	556	0.3802
	8	0.832	0.757	3.437	556	0.0006	0.647	8.567	556	0.0000
	16	0.864	0.731	6.437	556	0.0000	0.521	16.643	556	0.0000
	32	0.864	0.709	8.141	556	0.0000	0.381	25.276	556	0.0000
ND5	4	0.538	0.836	21.020	1537	0.0000	0.808	18.996	1537	0.0000
	8	0.557	0.815	17.573	1537	0.0000	0.717	10.92	1537	0.0000
	16	0.580	0.797	14.956	1537	0.0000	0.619	2.718	1537	0.0066
	32	0.590	0.781	13.385	1537	0.0000	0.504	5.978	1537	0.0000
ND6 1+2	4	0.600	0.783	5.673	316	0.0000	0.755	4.812	316	0.0000
	8	0.599	0.736	4.005	316	0.0001	0.627	0.804	316	0.4222
	16	0.604	0.687	2.380	316	0.0179	0.480	3.570	316	0.0004
	32	0.620	0.685	1.824	316	0.0691	0.355	7.475	316	0.0000
ND6 3	4	0.861	0.778	2.158	167	0.0323	0.779	2.137	167	0.0340
	8	0.897	0.741	4.512	167	0.0000	0.652	7.101	167	0.0000
	16	0.911	0.628	9.360	167	0.0000	0.453	15.127	167	0.0000
	32	0.925	0.705	7.846	167	0.0000	0.404	18.566	167	0.0000
ND6	4	0.681	0.796	4.512	482	0.0000	0.762	3.171	482	0.0016
	8	0.698	0.752	2.061	482	0.0399	0.640	2.197	482	0.0285
	16	0.698	0.721	0.877	482	0.3807	0.512	7.148	482	0.0000
	32	0.712	0.703	0.341	482	0.7331	0.378	12.789	482	0.0000

b) Interpretation of results.

	Significant Difference (p-value)	
	Yes	No
Iss < Iss.c	Little saturation	Substantial saturation
Iss > Iss.c	Useless sequences	Very poor for phylogenetic
