# Supplementary material for: Phylomitogenomics of two Neotropical species of long-legged crickets Endecous Saussure, 1878 (Orthoptera: Phalangopsidae)
Source: Genet Mol Biol. 2024 Apr 15;46(3 Suppl 1):e20230144. doi: 10.1590/1678-4685-GMB-2023-0144 (PMC11034622; doi:10.1590/1678-4685-GMB-2023-0144)
Supplement: Table S5 - [file 1415-4757-GMB-46-03-s1-e20230144-s5.pdf]

Genetics and Molecular Biology

https://doi.org/10.1590/1678-4685-GMB-2023-0144

Supplementary Material to "Phylomitogenomics of two Neotropical species of long-legged crickets *Endecous* Saussure, 1878 (Orthoptera: Phalangopsidae)"

Table S5 – Codon usage analysis of *Dianemobius nigrofasciatus*, *Endecous chape* and *E. onthophagus* mitogenomes.

Codon	Count	RSCU	Codon	Count	RSCU	Codon	Count	RSCU
UUU(F)	253/244/238	1.55/1.55/1.47	CCC(P)	26/20/26	0.76/0.58/0.72	GAU(D)	57/58/59	1.73/1.57/1.64
UUC(F)	74/71/86	0.45/0.45/0.53	CCA(P)	42/41/38	1.23/1.20/1.06	GAC(D)	9/16/13	0.27/0.43/0.36
UUA(L2)	336/354/299	3.45/3.64/3.03	CCG(P)	2/2/4	0.06/0.06/0.11	GAA(E)	81/67/64	1.71/1.70/1.64
UUG(L2)	70/57/89	0.72/0.59/0.90	ACU(T)	66/84/79	1.58/1.69/1.63	GAG(E)	14/12/14	0.29/0.30/0.36
CUU(L1)	69/80/86	0.71//0.82/0.87	ACC(T)	24/31/27	0.57/0.62/0.56	UGU(C)	41/40/40	1.78/1.82/1.95
CUC(L1)	30/25/24	0.31/0.26/0.24	ACA(T)	71/79/84	1.70/1.59/1.73	UGC(C)	5/4/1	0.22/0.18/0.05
CUA(L1)	73/64/85	0.75/0.66/0.86	ACG(T)	6/5/4	0.14/0.10/0.08	UGG(W)	13/7/11	1.00/1.00/1.00
CUG(L1)	7/4/9	0.07/0.04/0.09	GCU(A)	71/81/83	2.04/1.87/1.90	CGU(R)	15/22/21	0.59/1.05/0.98
AUU(I)	334/288/252	1.70/1.56/1.52	GCC(A)	15/29/30	0.43/0.67/0.69	CGC(R)	5/3/5	0.20/0.14/0.23
AUC(I)	53/73/75	0.27/0.40/0.45	GCA(A)	48/56/56	1.38/1.29/1.28	CGA(R)	27/29/28	1.07/1.38/1.31
AUA(I)	201/193/170	1.031.05/1.03	GCG(A)	5/7/6	0.14/0.16/0.14	CGG(R)	8/6/8	0.32/0.29/0.38
AUG(M)	65/48/57	1.00/1.00/1.00	UAU(Y)	128/137/124	1.65/1.72/1.51	AGU(S1)	19/39/32	0.43/0.85/0.72
GUU(V)	83/80/71	1.87/1.71/1.31	UAC(Y)	27/22/40	0.35/0.28/0.49	AGC(S1)	3/4/10	0.07/0.09/0.23
GUC(V)	10/11/17	0.22/0.24/0.31	CAU(H)	54/66/62	1.44/1.53/1.48	AGA(R)	89/60/61	3.51/2.86/2.86
GUA(V)	59/72/95	1.33/1.54/1.76	CAC(H)	21/20/22	0.56/0.47/0.52	AGG(R)	8/6/5	0.32/0.29/0.23
GUG(V)	26/24/33	0.58/0.51/0.61	CAA(Q)	59/64/66	1.79/1.71/1.69	GGU(G)	55/68/81	0.96/1.12/1.32

UCU(S2)	97/104/101	2.17/2.27/2.29	CAG(Q)	7/11/12	0.21/0.29/0.31	GGC(G)	11/7/12	0.19/0.12/0.20
UCC(S2)	54/34/29	1.21/0.74/0.66	AAU(N)	138/135/147	1.53/1.67/1.72	GGA(G)	135/135/115	2.36/2.22/1.87
UCA(S2)	86/92/92	1.93/2.01/2.08	AAC(N)	42/27/24	0.47/0.33/0.28	GGG(G)	28/33/38	0.49/0.54/0.62
UCG(S2)	9/2/1	0.20/0.04/0.02	AAA(K)	67/61/56	1.46/1.51/1.44			
CCU(P)	67/74/76	1.96/2.16/2.11	AAG(K)	25/20/22	0.54/0.49/0.56			

[a]Data of genes are given as *D. nigrofasciatus*/ *E. chape*/ *E. onthophagus*.
