# Supplementary material for: Phylomitogenomics of two Neotropical species of long-legged crickets Endecous Saussure, 1878 (Orthoptera: Phalangopsidae)
Source: Genet Mol Biol. 2024 Apr 15;46(3 Suppl 1):e20230144. doi: 10.1590/1678-4685-GMB-2023-0144 (PMC11034622; doi:10.1590/1678-4685-GMB-2023-0144)
Supplement: Figure S1 - [file 1415-4757-GMB-46-03-s1-e20230144-s6.pdf]

Genetics and Molecular Biology

https://doi.org/10.1590/1678-4685-GMB-2023-0144

Supplementary Material to "Phylomitogenomics of two Neotropical species of long-legged crickets *Endecous* Saussure, 1878 (Orthoptera: Phalangopsidae)"

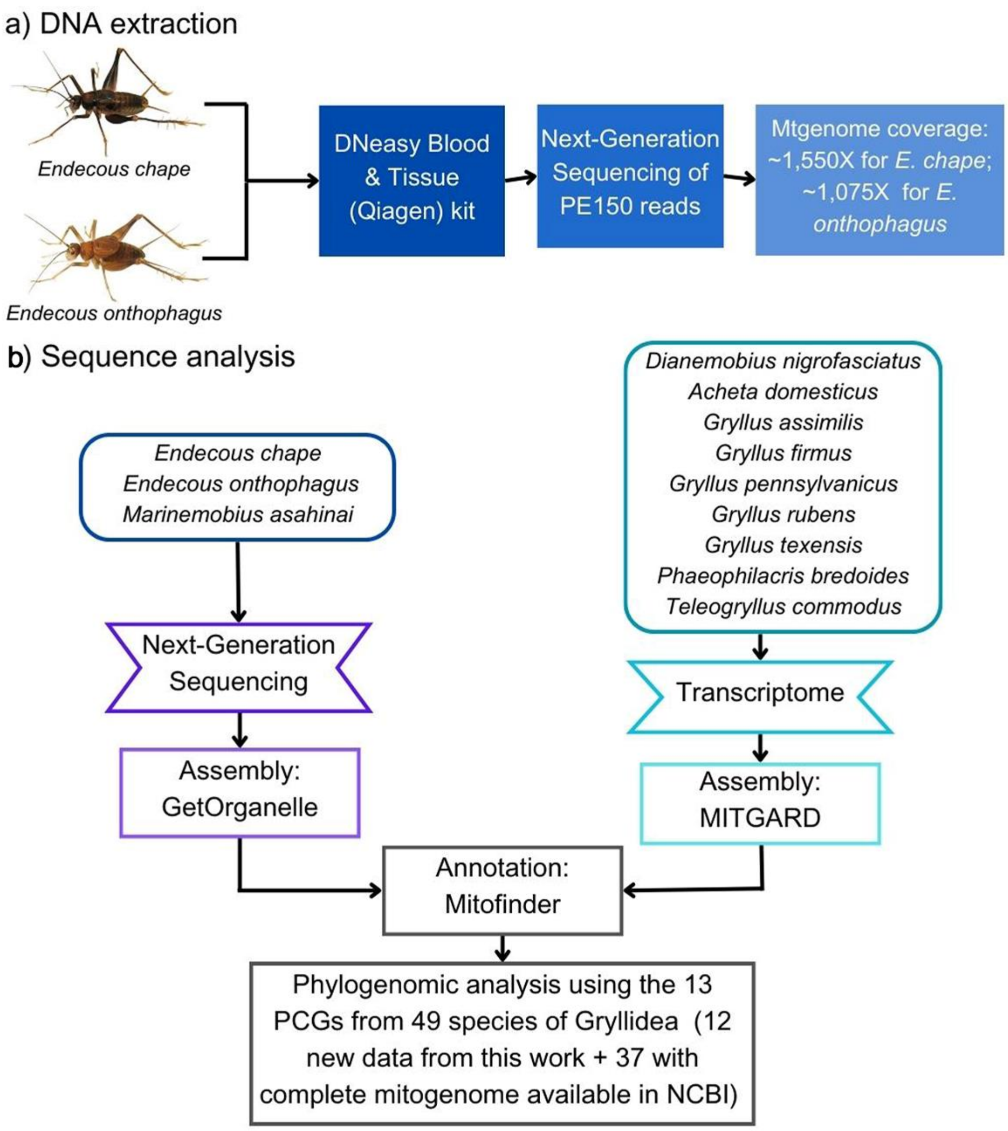

Figure S1 – Scheme of new data origin and methodology to obtain mitochondrial sequences from Next-Generation Sequencing (NGS) and Transcriptomes data for phylogenomic analysis. a) DNA extraction of *Endecous chape* and *E. onthophagus* using a Qiagen kit, performing NGS, and obtaining more than 1,000x of mitogenome coverage; b)

Mitochondrial sequences analysis applied in both *Endecous* and ten species with raw data available in NCBI. For NGS data, assembling was performed using the GetOrganelle pipeline, and for Transcriptomes the MITGARD, then annotation of the 13 PCGs was done with Mitofinder. Then the phylogenomic analysis of the infraorder Gryllidea was compound with 12 species from this work and 37 with complete mitogenome from NCBI.
