# Supplementary material for: Phylomitogenomics of two Neotropical species of long-legged crickets Endecous Saussure, 1878 (Orthoptera: Phalangopsidae)
Source: Genet Mol Biol. 2024 Apr 15;46(3 Suppl 1):e20230144. doi: 10.1590/1678-4685-GMB-2023-0144 (PMC11034622; doi:10.1590/1678-4685-GMB-2023-0144)
Supplement: Figure S3 - [file 1415-4757-GMB-46-03-s1-e20230144-s8.pdf]

Genetics and Molecular Biology

https://doi.org/10.1590/1678-4685-GMB-2023-0144

Supplementary Material to "Phylomitogenomics of two Neotropical species of long-legged crickets *Endecous* Saussure, 1878 (Orthoptera: Phalangopsidae)"

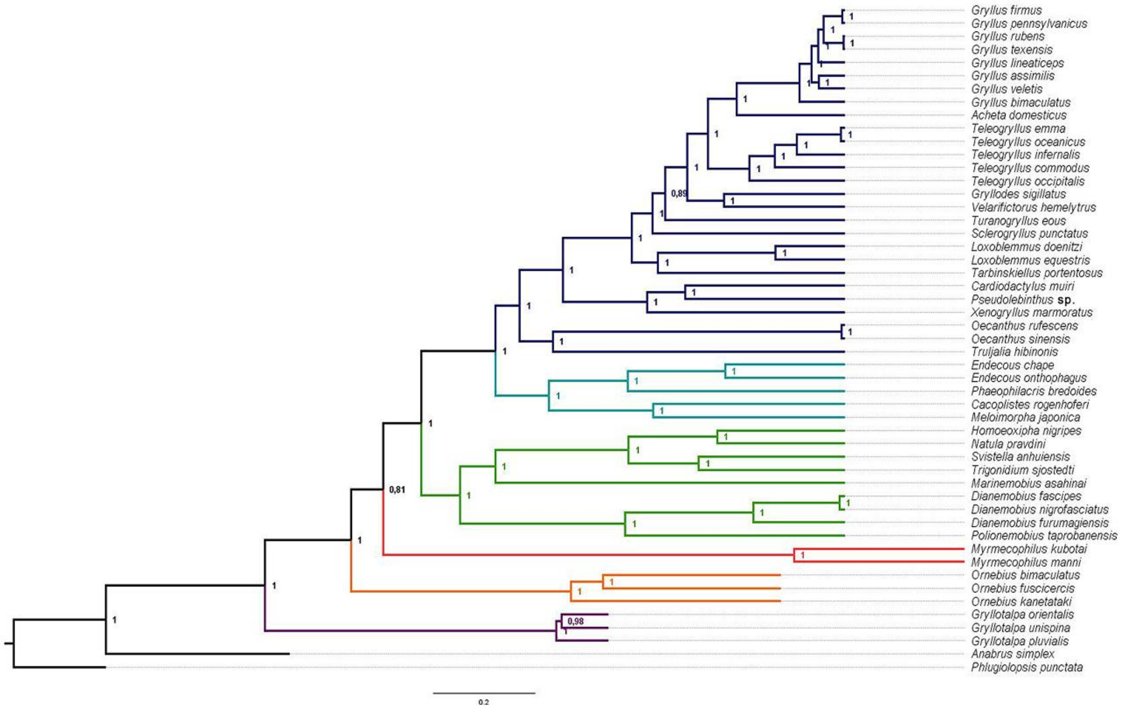

Figure S3 - Phylogenomic tree of infraorder Gryllidea inferred with the 13 PCGs using the Bayesian Inference (BI) method. Values next to the nodes indicate the posterior probabilities. Each branch color indicates the species family: purple Gryllotalpidae, orange Mogoplistidae, red Myrmecophilidae, green Trigonidiidae, dark green Phalangopsidae, and dark blue Gryllidae.
